# Complete Androgen Insensitivity Syndrome: A Rare Case of Disorder of Sex Development

**DOI:** 10.1155/2013/232696

**Published:** 2013-02-27

**Authors:** Alfonsa Pizzo, Antonio Simone Laganà, Irene Borrielli, Nella Dugo

**Affiliations:** ^1^Department of Pediatric, Gynecological, Microbiological and Biomedical Sciences, University of Messina, Via C. Valeria 1, 98125 Messina, Italy; ^2^Department of Gynecology and Obstetrics, Campus Bio-Medico, Via Álvaro del Portillo 200, 00128 Roma, Italy

## Abstract

Androgen Insensitivity Syndrome (AIS) could be considered as a disease that causes resistance to androgens actions, influencing both the morphogenesis and differentiation of the body structures, and systems in which this hormone exerts its effects. It depends on an X-linked mutations in the Androgen Receptor (AR) gene that express a variety of phenotypes ranging from male infertility to completely normal female external genitalia. The clinical phenotypes of AIS could vary and be classified into three categories, as complete (CAIS), partial (PAIS), and mild (MAIS) forms, according to the severity of androgen resistance. We will describe a case of CAIS in a 16-year-old patient.

## 1. Background

Androgen Insensitivity Syndrome (AIS) could be considered as a disease that causes resistance to androgens actions, influencing both the morphogenesis and differentiation of the body structures and systems in which this hormone exerts its effects. It depends on an X-linked mutations in the Androgen Receptor (AR) gene that express a variety of phenotypes ranging from male infertility to completely normal female external genitalia. The first who described this syndrome was John Morris [1], but we have to wait until 1989 to define the exact location of the human AR gene on Xq11-12 locus [2] and to have proof that is caused by mutations in this gene [3]. AR is expressed from 8 weeks of gestation: in male embryo, testes begin to secrete testosterone at 9 and peak its production at 11 and 18 weeks, stimulating differentiation of the Wolffian duct system into epididymis, vas deferens, and seminal vesicles. A more powerful androgen, dihydrotestosterone, originates from action of the enzyme 5alpha-reductase type 2 on testosterone and stimulates differentiation of the masculine primordial external genitalia [4]. The clinical phenotypes of AIS could vary and be classified into three categories, as complete (CAIS), partial (PAIS), and mild (MAIS) forms, and in seven grades, according to the severity of androgen resistance, like the one evidenced by Quigley et al. [5]. In particular, CAIS is characterized by a short blind ending vagina, absence of Wolffian duct-derived structures like epididymides, vas deferens and seminal vesicles, and absence of prostate. According to many authors [6–8], in CAIS the presence of structures derived from Müllerian ducts is reported very seldom. Clinical features of this syndrome include a totally female aspect from birth onwards. One important feature that helps to address correct diagnosis is that at puberty breast develops regularly, while there is scarce or absent development of pubic and axillary hair [5]. Diagnosis of CAIS could be very early, for example, when the mother underwent an amniocentesis that reported a 46,XY karyotype, and on the contrary obstetrics ultrasound or clinical evidence at birth showed the presence of female external genitalia [9, 10]. Another clinical element that could address the diagnosis of CAIS is the development of monolateral or bilateral inguinal hernia in the apparently female patient [11]. Patients with PAIS show very different clinical phenotype that depend on the severity of undervirilization. According to Quigley et al. [5], there are five grades of PAIS: in the first one there is normal female genital phenotype, with androgen-dependent pubic and/or axillary hair development at puberty; in the second grade, there is a female phenotype with mild clitoromegaly or small degree of posterior labial fusion; in the third grade, there are undifferentiated phallic structures intermediate between clitoris and penis, and the urogenital sinus presents perineal orifice and labioscrotal folds; the fourth grade is a predominantly male phenotype with perineal hypospadias, small penis, cryptorchidism or bifid scrotum; the fifth and last grade presents isolated hypospadias and/or micropenis. The clinical features of the last described PAIS forms are very similar to the MAIS: in this form, in fact, they could be coronal hypospadias or a prominent midline raphe of the scrotum [12]. At puberty, MAIS patients could have alteration in the spermatogenesis and fertility, but not in all cases [13, 14], while it is common to find impotence and gynecomastia. Endocrine features of CAIS and PAIS are the same: we could observe normal or overproduced serum Luteinizing Hormone (LH) and Testosterone (T) during the first three months of life. After this, LH and T levels are in the normal range until the puberty [15]. Then, at the puberty, we will find elevated serum levels of T and of LH, due to the androgen insensitivity and the consequent lack of negative feedback exerted by sex hormone on hypothalamus and hypophysis. Testosterone becomes itself target of aromatase, and so this enzyme coverts it in estrogens: for this reason CAIS patients present with higher estrogens levels than normal male and have good development of breast. Moreover, in patients with AIS, Anti-Müllerian Hormone (AMH) concentration is normal as the secretion and function of Sertoli and Leyding cells are not impaired [4]. In the following we describe a case of CAIS.

## 2. Subject and Method

### 2.1. Patient

The patient was 16 years old, with female habitus and voice, and normal intellectual function. Informed parental consent, patient consent, and approval by the Hospital Ethics Committee were obtained before initiating the report.

### 2.2. Clinical Features

The patient was sent to our observation by a provincial ambulatory service of gynecology and obstetrics, with diagnosis of hypergonadotropic hypogonadism. Previous exams showed elevated blood gonadotropins levels and infantile internal genitalia. At general physical examination, we found adipose tissue well represented, poor body hair, and hypotrophic breasts. The gynecological examination evidenced infantile external genitalia and viable vagina. Considering the suspected diagnosis of hypergonadotropic hypogonadism, we administered Hormone Replacement Therapy with oral estroprogestinics. At the follow-up after three months, the patient referred that there had been no change in primary amenorrhea. For this reason, we decided to repeat the hormonal and ultrasound examinations, obtaining the following results.

## 3. Laboratory Analysis

The hormonal levels were the following (Table 1).

### 3.1. Diagnosis: Ultrasound, Karyotype, and Laparoscopy

We performed transabdominal ultrasound that evidenced absence of uterus and of structures related to the ovaries in the area where it is commonly found, and then we determined a “male” 46,XY karyotype. After communicating the results to the parents (who preferred not to inform the patient), we decide to perform laparoscopy (which confirmed the results described by ultrasound) and to take biopsies of testicular-like tissue held in the pelvic cavity. Histological examination of biopsy tissue showed testicular parenchyma, seminiferous tubules of smaller size than the norm, and agenesis of the germinal epithelium. For this reason, considering the high incidence of malignant degeneration of the gonads held in the abdominal cavity, we addressed the patient to a bilateral gonadectomy.

### 3.2. Histological Examination

The histological examination of the removed gonads enlightened presence of spermatic cord and epididymal-didymus parenchyma. With reference to testicular tissue, we highlighted seminiferous tubules arranged in irregular lobular formations, for a process of interstitial and peritubular fibrosis. The tubular spaces were widely devoid of germ epithelium with the presence of immature Sertoli's cells in vacuolar degeneration, marked vascular congestion of parenchymal and hilar vessels. Moreover, there were associated findings of congestion and interstitial fibrosis of the funiculars sections and of the epididymal intertubular connective tissue.

## 4. Conclusion

All these findings were consistent with Complete Androgen Insensitivity Syndrome. Therefore, the patient was referred to a hormone replacement therapy. Parents preferred not to disclose the diagnosis to the daughter, to whom it was explained that, due to the absence of the uterus and ovaries, hormone therapy was needed but could not resolve nor amenorrhea nor infertility. This news was so devastating for a young psychologically female, who hoped to solve her problems in other ways.

## Figures and Tables

**Table 1:** Hormonal levels.

Analyte	Value
Triiodothyronine (T3)	1.15 ng/mL
Tetraiodothyronine (T4)	9.78 ug/dL
Thyroid stimulating hormone (TSH)	2.26 uUl/mL
Free triiodothyronine (FT3)	3.96 pg/mL
Free tetraiodothyronine (FT4)	20.55 pm/L
Thyroxine-binding globulin (TBG)	17.0 ug/mL
Follicle stimulating hormone (FSH)	58.51 mLU/mL
Luteinizing hormone (LH)	29.73 mLU/mL
Estradiol (E2)	8.33 pg/mL
Progesterone (PG)	0.92 ng/mL
Prolactin (PRL)	421.20 mLU/L
Sex hormone binding globulin (SHBG)	15.80 nmol/L
17- hydroxyprogesterone (17-OHPG)	3.90 ng/mL
Dehydroepiandrosterone-sulfate (DHEA-S)	262.00 ug/dL
Thymic serum factor (FTS)	8.20 pg/mL
Testosterone (T)	79.64 ng/dL
Delta-4 androstenedione (ASD)	5.76 ng/mL
Cortisol (CORT)	29.10 microg/dL
C-peptide (C-PEP)	1.89 ng/mL
Growth hormone (GH)	5.00 ng/mL
